# Sintilimab as maintenance treatment for local/regional recurrent esophageal squamous carcinoma after concurrent chemoradiotherapy: a single-arm Ib/II phase study

**DOI:** 10.3389/fimmu.2023.1193394

**Published:** 2023-05-31

**Authors:** Chengxin Liu, Hongfu Sun, Wei Huang, Zhongtang Wang, Chengrui Fu, Dan Han, Qian Zhao, Xue Wu, Baosheng Li

**Affiliations:** ^1^ Shandong Cancer Hospital, Cheeloo College of Medicine, Shandong University, Jinan, Shandong, China; ^2^ Department of Radiation Oncology, Shandong Cancer Hospital and Institute, Shandong First Medical University and Shandong Academy of Medical Sciences, Jinan, Shandong, China; ^3^ College of Clinical Medicine, Southwest Medical University, Luzhou, Sichuan, China

**Keywords:** ESCC, Local/regional recurrent, CCRT, Immunotherapy, Clinical study

## Abstract

**Background:**

Esophageal cancer (EC) is an aggressive neoplasm of the gastrointestinal tract that is usually treated with a combination of chemotherapy, radiotherapy (RT), and/or surgery, according to disease status. Despite the availability of multimodal therapeutic strategies, local recurrence is frequently observed. However, there is no standard treatment or promising therapeutic approach for local recurrence or metastatic esophageal carcinoma after the RT. This study tended to investigate the efficacy and safety of sintilimab maintenance after concurrent chemoradiotherapy (CCRT) for local/regional recurrent esophageal squamous carcinoma.

**Methods:**

This study was a single-arm, phase Ib/II trial conducted in a single site in China. Patients previously radically treated (surgery or CCRT), histologically confirmed, local or regional recurrence esophageal squamous carcinoma, qualified for the study design, were treated with 25–28 times radiotherapy plus raltitrexed once every 3 weeks for up to two cycles. Patients who have not progressed after CCRT received sintilimab as maintenance once every 3 weeks up to 1 year. Primary endpoints were overall survival (OS) and safety. Secondary endpoints were progression-free survival (PFS), objective response rate (ORR), and duration of response (DOR).

**Results:**

Between September 2019 and March 2022, in a total of 36 enrolled patients, 34 pts completed CCRT. Three patients excluded due to violation of the exclusion criteria (1 pt) and consent withdrawal (2 pts). Finally, 33 pts were included in the final analysis, in which 3 pts had disease progression, and the remaining 30 entered maintenance therapy with sintilimab. The median follow-up time was 12.3 months. Median OS was 20.6 months (95%CI 10.5–NA) and the 1-year OS rate was 64%. Median PFS was 11.5 months (95%CI 5.29–21.3) and the 1-year PFS rate was 43.6%. The ORR was 63.6% (95%CI 44.6–77.8), including 2 cases of CR and 19 cases of PR. The DCR was 19.9%, the median DOR was 19.5 months, and the median TTR was 2.4 months. The rate of any grade TRAEs was 96.7%; ≥Grade 3 TRAE was 23.4%. The incidence of immune-related AE was 60%, most of which were grade 1–2, and only one case of thyroid-stimulating hormone increased was irAE with grade 3 or above.

**Conclusion:**

Sintilimab has shown promising clinical efficacy and a manageable safety profile as maintenance therapy after CCRT for local/regional recurrent esophageal squamous carcinoma. In addition, further confirmation from a large-scale real-world study is still needed.

## Introduction

Esophageal cancer (EC) is a major tumor, and its morbidity and mortality are at the forefront of malignant tumors. Esophageal squamous cell carcinoma accounts for more than 95% of cases in China, most of which are found in the middle and late stages (1). For such patients, synchronous (neoadjuvant) chemoradiotherapy and radical surgical treatment are standard strategies (2, 3). Local recurrence after radical treatment is the main failure mode, the local recurrence rate after radical chemoradiotherapy is as high as 47%, and the postoperative regional recurrence probability is also very high (4). Once recurrence or metastasis occurs, most patients die within 1 year (5).

For patients with recurrence after surgery, the 2016 Union for International Cancer Control (NCCN) guidelines recommend chemoradiotherapy as the standard treatment, and fluorouracil- or taxane-based chemoradiotherapy is recommended as the preferred regimen. However, due to the lack of evidence, there is no unified standard for the radiotherapy target area and dose (6). Neoadjuvant chemoradiotherapy followed by surgery improves the survival of patients with resectable locally advanced EC. However, approximately 10% of regional lymph node metastasis and 14% of local recurrence still occur. In addition, recurrence in the supraclavicular lymph node region is not uncommon (7). For these patients, salvage chemoradiotherapy can be used as a radical treatment (8). In terms of safety, even for patients who relapsed after neoadjuvant chemoradiotherapy, few safety events have been reported. In addition, according to the the chemoradiotherapy for esophageal cancer followed by surgery study (CROSS) study, most of the local recurrence occurred outside the radiotherapy field. It provides a high degree of safety guarantee for the treatment of chemoradiotherapy. However, in patients with recurrence after curative treatment, the response rate to treatment is approximately 30%, and the median PFS is only approximately 4 months. It is of great significance to explore a more effective treatment mode for recurrent esophageal squamous cell carcinoma.

In recent years, some studies have shown that radiotherapy or chemotherapy can enhance the efficacy of immune checkpoint inhibitors by destroying the genome of tumor cells and enhancing immunogenicity. CRT+ immune-maintenance therapy has become the standard treatment for locally advanced NSCLC. However, there is still a lack of evidence in the field of EC.

Based on the above theory and clinical practice, we conducted a phase Ib/II study of immunotherapy after chemoradiotherapy for recurrent EC, aiming to verify the efficacy and safety of this treatment mode, analyze the therapeutic effect, and explore the biomarkers related to the efficacy.

## Materials and protocols

### Patients

This study was a single-arm, phase Ib/II trial conducted in a single institute in China. Eligibility was defined as follows: 1) previously radically treated (surgery or CCRT), histologically confirmed local or regional recurrence esophageal squamous cell carcinoma; 2) at least one measurable lesion; 3) suitable for the design of this study, according to the researchers’ evaluation (if radiotherapy was included in the previous treatment, at least 8 months had passed since the end of the previous radiotherapy before enrollment); 4) age 18–75 years, who could understand and sign the content and risks of the clinical trial and signed informed consent; 5) Eastern Cooperative Oncology Group (ECOG) score 0 or 1, expected survival of more than 3 months; and 6) there was no obvious myelosuppression at the time of enrollment: hemoglobin ≥9g/dl, neutrophils ≥1.5 × 10^9^/L, and platelets ≥100 × 10^12^/L. This clinical trial was approved by the Drug Clinical Trial Ethics Committee of Shandong Cancer Hospital on 7 March 2019 (Approval No. SDZLEC2019-017-01). This trial is registered on Chinese Clinical Trial Registry as ChiCTR1900027161 (https://www.chictr.org.cn). Pre-treatment assessment included the following.

### Treatment and assessment

After screening, patients with esophageal squamous cell carcinoma who met the inclusion and exclusion criteria signed the informed consent form(ICF) and then received radiotherapy at all recurrent sites, with a dose of 1.8 Gray (Gy) in 25–28 and a total radiation dose of 45–50.4 Gy. On the first day of radiotherapy, one cycle of raltitrexed (3 mg/m^2^) was administered. The second cycle of chemotherapy was given on day 22 after chemotherapy. The efficacy was evaluated by CT or PET-CT within 4 weeks after radiotherapy. Patients without progressive disease after chemoradiotherapy entered the maintenance phase of sintilimab monotherapy. Sintilimab (200 mg) was injected every 3 weeks until intolerance or progressive disease. Sintilimab was used for no more than 1 year.

At the time of enrollment, all patients provided their past medical history, imaging data, complete and continuous physical examination, functional status score, blood routine, blood biochemistry, serum tumor markers, thyroid function, electrocardiogram, pulmonary function, and other general examination items. Patients should be examined by contrast-enhanced CT and a barium meal within 1 week before radiotherapy and 4 weeks after radiotherapy. The efficacy was evaluated according to RECIST v1.1. Endoscopic ultrasonography can be performed if necessary. From the maintenance treatment phase, blood routine and blood biochemistry were tested at least every 3 weeks. Tumor markers were tested at least every 4 weeks, with enhanced CT and a barium meal every 8 weeks.

Adverse events were collected and monitored from the end of chemoradiotherapy until 3 months after the last immunotherapy or death. Adverse events were graded according to the Common Terminology Criteria for Adverse Events, version 5.0. After treatment, patients should return to the hospital every 8–12 weeks for a follow-up, including symptoms, physical examination, hematological evaluation, and imaging evaluation. Patients were followed up by telephone every 4–8 weeks until death or until the end of the study.

### Endpoints

The primary endpoints were safety profile and overall survival (OS), defined as the time from the start of treatment to death from any cause. Secondary endpoints included progression-free survival (PFS) (the time from the start of treatment to the first failure at any site or death from any cause, whichever occurred first) and tumor response.

### Statistical analysis

Via SAS 9.2 software, all statistical analyses were conducted using a one-sided 0.05 hypothesis test, with 95% confidence interval. Unless otherwise stated, measurement data were statistically described as mean ± standard deviation or median (minimum and maximum). Counting data were statistically described by frequency (percentage). The Kaplan–Meier curve was used to estimate median OS and PFS. The statistical hypotheses of this study are as follows: H0 = 12, H1 = 19, α = 0.1, and β = 0.2. According to the dropout rate of 10%, the final sample size was 36 cases.

## Results

### Treatment tolerance and compliance

**Figure 1:**
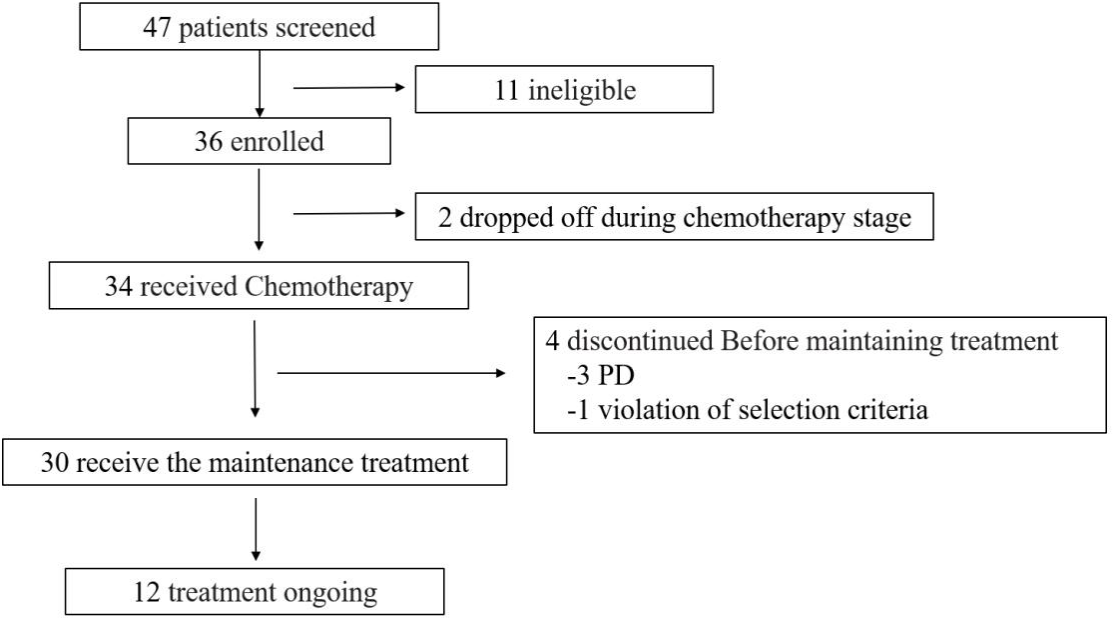
Trial profile.

From September 2019 to March 2022, in a total of 36 enrolled pts (median age 63, 95%CI:48–74 years old), 34 pts completed CCRT. Three patients excluded due to violation of the exclusion criteria (1 pt) and consent withdrawal (2 pts). Finally, 33 pts were included in the final analysis, all of which had complete baseline data and safety evaluation (**Figure 1**).

**Figure 2:**
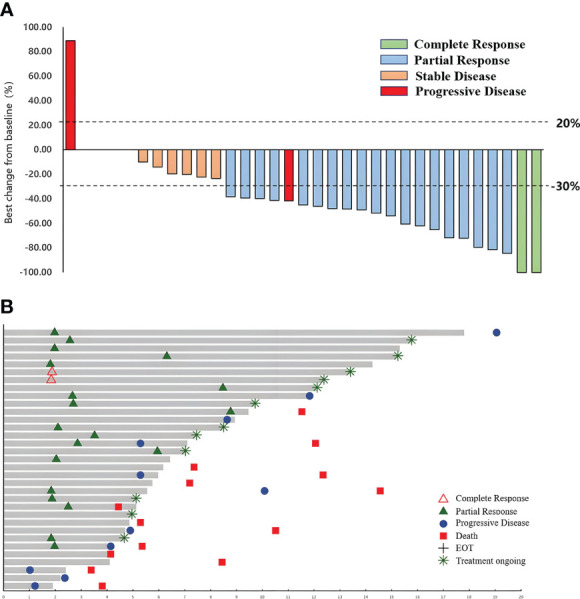
Best percentage change in the sum of diameters of target lesions from baseline **(A)** and time on treatment **(B)** in the efficacy analysis set.

After CCRT, 90.9% (30/33) of patients entered the sintilimab maintenance stage, and 3 patients had disease progression (**Figure 2B**). As of 1 September 2022, 12 pts were still receiving sintilimab treatment, while 18 pts ended. Among them, eight patients were excluded from the group after 1 year of treatment, seven patients progressed or died, and three patients were excluded from the group for other reasons.

### Patient characteristics

The median age of the 33 patients was 63 years (range 48–74 years), 87.9% (4/33) were men, 91% had an ECOG score of 1 (30/33), and smoking accounted for 54.5% (18/33). The follow-up treatment received by the subjects included two cases of reuse of other immunotherapy, two cases of radiotherapy, one case of particle implantation, five cases of chemotherapy, three cases of androtinib treatment, and two cases of optimal supportive treatment. The detailed characteristics of the patients are shown in **Table 1**.

**Table 1:** Baseline characteristics.

Characteristic	N = 33
Age, n	
Mean(SD)	62.6( ± 6.6)
Median	63(48, 76)
Gender, n(%)	
Male	29(87.9)
Female	4(12.1)
Smoking history, n(%)	
Yes	18(54.5)
No	15(45.5)
ECOG PS	
0	3(9.1)
1	30(90.9)
Disease stage, n(%)	
II	4(12.1)
III	16(48.5)
IV	13(39.4)
Previous treatment, n(%)	
Surgery	22(66.7)
Chemotherapy	25(75.8)
Radiotherapy	20(60.6)
M stage	
M0	20(60.6)
M1	13(39.4)
T stage, n(%)	
T0	11(33.3)
T1	2(6.1)
T3	10(30.3)
T4	1(3)
TX	9(27.3)
N stage, n(%)	
N0	7(21.2)
N1	19(57.6)
N2	6(18.2)
NX	1(3)

### Efficacy

**Figure 3:**
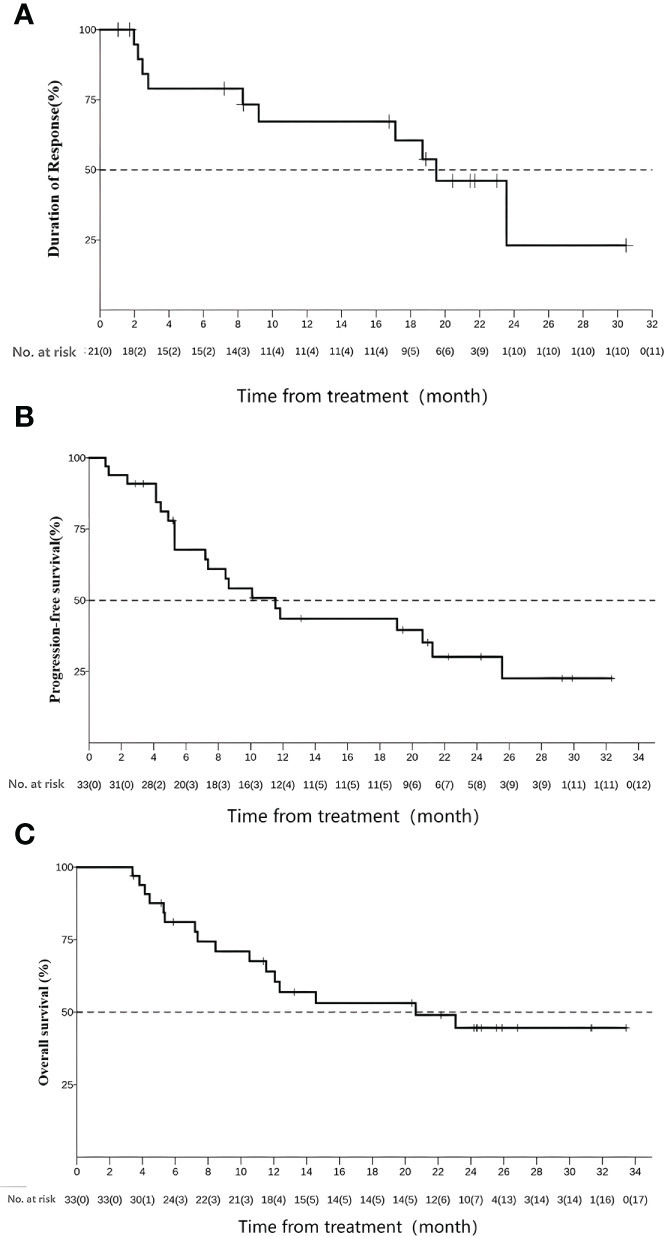
Duration of response **(A)**; progression-free survival (PFS) **(B)** and overall survival (OS) **(C)**.

As of 1 September 2022, the median OS follow-up duration reached 12.3 months (IQR 3.4–33.4) (**Figure 3A**). For the intention-to-treat population, the median OS was 20.6 months (95%CI 10.5–NA), 1-year and 2-year OS rates were 64% (95%CI 49–83.1) and 44.6% (95%CI 29.3–67.8), respectively (**Figure 3C**). The median PFS was 11.5 months (95%CI 5.29–21.3), and 1-year and 2-year PFS rates were 43.6% (95%CI 28.9–65.7) and 30.2% (95%CI 16.7–54.5), respectively (**Figure 3B**).

Among the 33 pts in this study, the objective response rate assessed by the investigator per RECIST 1.1 was 63.6% (95%CI 44.6–77.8), with 2 (6.1%) confirmed complete response (CR) and 19 (57.6%) confirmed partial response (PR).

The disease control rate (DCR) was 90.9% (95%CI: 76.4–96.9), and the median time to response (mTTR) was 2.4 months (**Figure 2A**).

**Figure 4:**
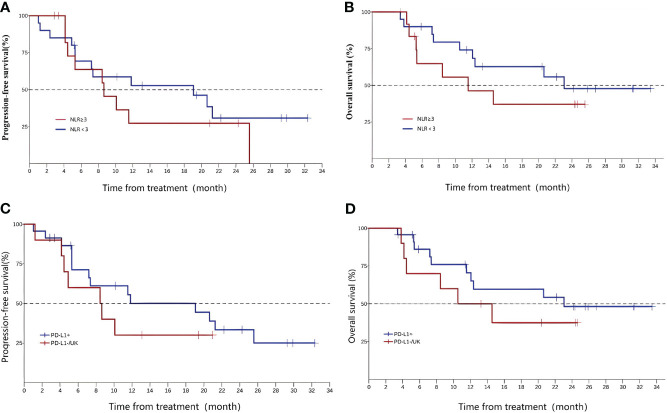
Neutrophil-to-lymphocyte ratio, PFS **(A)**, and OS **(B)**;programmed death-ligand 1 (PD-L1) expression(CPS, PD-L1+ vs PD-L1-/UK), PFS **(C)**, and OS **(D)**.

As is shown in Supplement, one-way ANOVA analysis showed that no factor influenced the OS. The *post hoc* analysis of the baseline neutrophil-to-lymphocyte ratio (NLR) of the subject is shown in **Figures 4A, B**. The median PFS was 8.64 months (95%CI 4.14, NA) for subjects with NLR≥3 and 19.06 months (95%CI 5.29, NA) for subjects with NLR < 3 at baseline; the median OS was 11.5 months (95%CI 4.44, NA) and 23.1 months (95%CI 10.51, NA), respectively. These data are shown in **Table 2**.

**Table 2:** Objective response and disease response.

Response	N = 33(%)
Complete response	2(6.1)
Partial response	19(57.6)
Stable disease	9(27.3)
Progressive disease	3(9.1)
ORR(95% CI)	63.6%(44.6%, 77.8%)
DCR(95% CI)	90.9%(76.4%, 96.9%)
mTTR	2.4m

In this study, the subjects voluntarily underwent programmed death-ligand 1 (PD-L1) expression testing, and a total of 23 cases were PD-L1 positive, 7 cases were negative, and 3 cases were unknown. According to the test results, patients were divided into the PD-L1-positive group (23 cases) and PD-L1-negative or unknown group (10 cases) for analysis. The mPFS of the two groups were 11.83 (5.29, 25.6) and 8.54 (1.23, NA), and the OS were 23.1 (11.53, NA) and 12.5 (3.81, NA), respectively (**Figures 4C, D**).

### Safety

A total of 29 patients (96.7%) experienced any grade treatment-related adverse events (TRAEs), most of which were grade 1–2. The incidence of grade 3 or higher adverse events was 23.3%. It mainly includes three lymphocyte count decreased, 1 hypoalbuminemia, 1 anemia, 1 Belom cell number decreased, 1 aspartate aminotransferase increased, 2 neutrophil count decreased, 1 platelet count decreased, and 1 thyroid-stimulating hormone increased. There were four cases of adverse events leading to drug withdrawal, including hypoparathyroidism, pneumonia, and upper gastrointestinal bleeding. Immune-related adverse events occurred in 60% of patients and were mostly grade 1–2. Only one case of grade 3 or above irAE occurred, which was the thyroid-stimulating hormone decreased. There was no AE incidence resulting in death (**Table 3**).

**Table 3:** ≥10% treatment-related adverse events and potential immune-related adverse events.

	N = 30(%)	Grade 1(%)	Grade 2(%)	Grade 3(%)	Grade 4(%)
≥10% treatment-related adverse events					
All	29(96.7)	29(96.7)	20(66.7)	5(16.7)	2(6.7)
Lymphocyte count decreased	26(86.7)	17(56.7)	6(20)	3(10)	0
Hypoalbuminemia	23(76.7)	20(66.7)	2(6.7)	0	1(3.3)
Anemia	20(66.7)	15(50)	4(13.3)	0	1(3.3)
Blood bilirubin elevation	15(50)	13(43.3)	2(6.7)	0	0
Lower creatinine	15(50)	15(50)	0	0	0
Belom cell number decreased	14(46.7)	6(20)	7(23.3)	0	1(3.3)
Hypoalbuminemia	13(43.3)	13(43.3)	0	0	0
Blood lactate dehydrogenase increased	12(40)	12(40)	0	0	0
Cordone phosphate kinases decreased	12(40)	12(40)	0	0	0
Aspartate aminotransferase decreased	11(36.7)	11(36.7)	0	0	0
Cholesterol high	11(36.7)	11(36.7)	0	0	0
Alanine aminotransferase increased	9(30)	7(23.3)	2(6.7)	0	0
Aspartate aminotransferase increased	9(30)	7(23.3)	1(3.3)	1(3.3)	0
Decreased trioxyticoine	9(30)	9(30)	0	0	0
Hyperglycemia	8(26.7)	8(26.7)	0	0	0
Neutrophil count decreased	7(23.3)	4(13.3)	1(3.3)	1(3.3)	1(3.3)
Cordone phosphate kinases increased	6(20)	5(16.7)	1(3.3)	0	0
Alanine aminotransferase decreased	6(20)	6(20)	0	0	0
Platelet count decreased	5(16.7)	4(13.3)	0	0	1(3.3)
Neutrophil count increased	5(16.7)	5(16.7)	0	0	0
Hypoparathyroidism	5(16.7)	5(16.7)	0	0	0
Thyroid-stimulating hormone increased	5(16.7)	4(13.3)	0	0	1(3.3)
Low-density lipoprotein increased	5(16.7)	5(16.7)	0	0	0
Uric acid decreased	5(16.7)	4(13.3)	1(3.3)	0	0
Platelet count increased	4(13.3)	4(13.3)	0	0	0
Fiber protein increased	4(13.3)	4(13.3)	0	0	0
Potential immune-related adverse events					
All	18(60)	16(53.3)	3(10)	0	1(3.3)
Decreased trioxyticoine	9(30)	9(30)	0	0	0
Thyroxine decreased	5(16.7)	5(16.7)	0	0	0
Thyroid-stimulating hormone increased	4(13.3)	3(10)	0	0	1(3.3)
Hypervascular calcium protein increased	4(13.3)	3(10)	1(3.3)	0	0
Hypoparathyroidism	2(6.7)	1(3.3)	1(3.3)	0	0
Pneumonia	1(3.3)	0	1(3.3)	0	0
Malaise	1(3.3)	1(3.3)	0	0	0
Thyroid-stimulating hormone decreased	1(3.3)	1(3.3)	0	0	0
Type B sodium urine peptide anterior body increased	1(3.3)	1(3.3)	0	0	0
Fever	1(3.3)	1(3.3)	0	0	0

## Discussion

As was shown, sintilimab maintenance therapy after CCRT provided significant and clinically meaningful improvements in OS, PFS, and the ORR compared with the historical data and promising antitumor activity in patients with local/regional recurrence of advanced esophageal squamous carcinoma. Most TRAEs were manageable, and no new safety signals were observed. The use of sintilimab after concurrent radiotherapy and chemotherapy does play a crucial role in the consolidation treatment stage. It can be intuitively demonstrated through the following historical data and regression studies. Our study included patients of previously radially treated (surgery or CCRT). For patients with recurrence after CCRT, Chinese data show a 1-year OS rate of 51% and a median survival of 12 months (9). Compared to the above data, our research results (1-year OS rate of 64%, median survival time of 20.6 months) are more encouraging. For patients with recurrent ESCC after radical surgery, a meta-analysis showed a 1-year OS rate of 67% and a median survival of 25 months, which is basically equivalent to our data (10). Moreover, the “long tail effect” of immune maintenance therapy has been preliminarily demonstrated in this study, and we are confident that the 3-year or even 5-year OS of this study will be the highest among all reports.

The risk factors for serious complications of secondary radiotherapy include Carlisle score ≤ 70, total dose > 100 Gy, concurrent chemoradiotherapy (CCRT), and age < age 60, Stage T4 (11). In this study, we choose 50.4 Gy as the radical dose in this study according to the two clinical studies of RT0G8501 and RTOG9405 (12, 13). Raltitrexed and fluorouracil act on the same target enzyme, and 5-FU presented critical chemotherapy-induced cardiotoxicity. Given the cardiovascular toxicity, raltitrexed was used in CCRT treatment, a specific and selective inhibitor of thymidine synthetase, which can be converted into polyglutamate by folic acid carrier and stored in cells. However, the incidence of III–IV adverse reactions in the raltitrexed-based CCRT was relatively low, especially in the aspects of cardiotoxicity events and esophagitis, which was significantly lower than that of the 5-FU regimen. The ORIENT-15 study of sintilimab in first-line EC showed that the TRAEs and Grade 3–5 TRAEs of sintilimab combined with chemotherapy were significantly lower than those of the chemotherapy group: 54.3% vs. 90.8% and 20.2% vs. 39.1%, suggesting good safety of sintilimab (14). As sintilimab showed good antitumor activity and a manageable safety profile in previous studies, sintilimab was selected for immune-maintenance therapy after CCRT (15). In this study, the incidence of all TRAEs was 98.1%, of which the incidence of ≥ grade 3 TRAEs was 23.3%. The incidence of immune-related adverse events was 60%, and only one patient developed grade ≥3 irAE. The safety data of this study were similar to those of ORIENT-15. Also in Keynote-590 (16), the incidence of TRAEs was 98.4%, and the incidence of ≥ grade 3 TRAE was 71.9%. In checkmate-648 (17), the rate of TRAEs was 96%, and the rate of grade ≥3 TRAE was 47%. As compared with other PD-1 inhibitor classes, sintilimab had a manageable safety profile, and the incidence of safety events in this study was within expectations. In addition, in a meta-analysis of lung cancer, it was found that the toxicity and side effects of sindilimab were the lowest among all PD-1 inhibitors. Therefore, we chose sindilimab for immune maintenance therapy after CCRT (18).

The OS benefit (20.6 months) and PFS benefit (11.5 months) were observed in patients accepting sintilimab maintenance therapy after CCRT, and the ORR reached 63.6%, the median DOR was 19.5 months. It may relate to that radiation increasing the probability of tumor recognition by the host immune system, activating the cGAS-STING pathway to trigger an immune response, and reconstructing the tumor microenvironment mechanism. Kelly et al. reported that neoadjuvant chemoradiotherapy may upregulate PD-L1 expression and thus improve the response to the PD-1/L1 antibody (19). The probable mechanism and study results provided the primary evidence for the rationality of immunotherapy plus a CCRT regimen.

Immune checkpoint inhibitors have changed the treatment landscape of tumors in recent years. In the phase III PACIFIC trial (20), 709 patients were randomized (2:1) to receive either adjuvant durvalumab or placebo every 2 weeks for up to 12 months after the completion of CCRT (two cycles of platinum-based chemotherapy and 54– 66 Gy radiotherapy). Consolidation with durvalumab prolongs long-term survival of patients with unresectable stage III non-small cell lung cancer whose disease has not progressed after concurrent chemotherapy and radiotherapy. The single-arm phase II LUN 14-179 trial (n ¼ 93) evaluated 1 year of adjuvant pembrolizumab after CCRT in patients with unresectable NSCLC and met its primary endpoint: time to metastatic disease or death was 30.7 months [95% CI 18.7 months–not reached] (21).

EC is a highly immunogenic tumor (22). The phase III clinical studies KEYNOTE-181, ATTRACTION-3, and ESCORT study of EC have confirmed that immunotherapy monotherapy can be used as the standard second-line treatment for EC (23–25). The ORIENT-2 study of sintilimab in the second-line treatment of EC also confirmed that its single-agent efficacy was superior to that of chemotherapy drugs such as paclitaxel or irinotecan. The median OS of the sintilimab group and chemotherapy group was 7.2 and 6.2 months, respectively, and the 12-month OS rate was 37.4% and 21.4%, respectively. Sintilimab has shown encouraging antitumor efficacy (26). However, less than 1 year of OS and unimproved PFS suggest that single immunotherapy cannot meet the treatment requirements for second-line EC. In the combination therapy, the ORR rate of the new immunostimulators anti TIGIT antibody (tiragolumab) and anti-PDL1 antibody (atezolizumab) in the treatment of second-line EC was 27.8%, while in the combination therapy of immune combined anti vascular therapy, the ORR rate of camrelizumab and apatinib was 34.6%. Our ORR rate reached 63.6%, which is significantly higher than the above two studies (27, 28). In addition, the CAP 02 study showed a PFS of 7.5 months and an OS of 15.8 months. Compared to our PFS of 11.5 months and OS of 20.6 months, our results were also slightly better. The CAP02 study used camrelizumab for maintenance therapy, while we used sindilimab for maintenance therapy, which indirectly reflects that sindilimab seems to have a better maintenance therapy effect than camrelizumab. Moreover, immunotherapy has also been used in many ongoing studies for different neoadjuvant immunotherapy as well (29). In neoadjuvant chemotherapy combined with immunotherapy, the pCR rate of the TD-NICE study reached 50% (30), and the postoperative pCR rate was 42.5% (31); furthermore, the KEYSTONE-001 study and the ESPRIT study pCR rate were 41.4% and 35%, respectively (32, 33). The above studies preliminarily provided the tolerable safety profile and clinical feasibility of neoadjuvant immunotherapy for EC. However, whether the high pCR rate can be translated into survival benefits needs to be further investigated, and the long-term postoperative survival results still need to be continuously accumulated and verified in the future.

The different therapies of neoadjuvant or adjuvant treatment using CRT plus immunotherapy are developing rapidly, but there is still currently a lack of biomarkers to accurately predict the efficacy of EC to guide perioperative immunotherapy. In addition, immunotherapy-related adverse reactions (irAEs) should be vigilant and closely monitored, and the update of multidisciplinary treatment concepts and strategies will allow more patients to benefit from immunotherapy.

Ng et al. has proven that the NLR before radiotherapy is a prognostic indicator of oropharyngeal cancer, and OS is higher in patients with NLR < 3 than in patients with NLR ≥ 3 (5-year OS 85 vs. 74%) (34). The similar phenomenon may occur in EC patients. ORIENT-2 (24), a study of sintilimab in the second-line treatment of EC, also analyzed the efficacy of patients with different NLRs and obtained similar results. In this study, the NLR was divided into two groups according to the cutoff value of 3, and the results showed that the median PFS and median OS of the NLR ≤ 3 group were better than those of the NLR > 3 group. However, due to the limited sample size of this study, it cannot prove that patients with NLR < 3 may have better treatment effects with sintilimab, and this conclusion still needs to be supported by data.

Multiple studies of immune checkpoint inhibitors have confirmed that the efficacy of PD-L1-positive patients is better than that of PD-L1 negative patients in different tumor types. Li ZC et al. conducted a meta-analysis of multiple phase III clinical trials in EC; subgroup analyses suggested significant OS advantage in PD-L1 tumor-positive score (TPS) ≥ 10% groups and obviously longer PFS in the PD-L1 combined positive score (CPS) ≥ 10 groups (35). In this study, according to PD-L1 expression, the patients were divided into the PD-L1+ group (PD-L1 CPS > 1) and PD-L1-/UK group (PD-L1 CPS ≤ 1 or PD-L1 expression unknown) for analysis. The results showed that the median PFS and median OS of the PD-L1+ group were significantly better than those of the other group, and the results were consistent with previous studies.

This clinical study still has some limitations: first, as a phase II single-arm, single-center clinical study, the number of included 36 patients is not convincing enough. Secondly, as the tumor tissue of patients was difficult to obtain, further comprehensive detection of PD-L1, TMB, or other biomarkers cannot be conducted, and a detailed subgroup analysis cannot be conducted either. The exception is that, due to the epidemic influence in China in 2022, part of patients showed poor compliance and thus cannot take the examinations during the follow-up according to the protocol, which had impact on the results to some extent.

## Conclusion

In conclusion, sintilimab maintenance therapy after CCRT can be used as an effective treatment for local relapsed esophageal squamous cell carcinoma with good safety. Further large-scale clinical studies are needed to support the evidence.

## Data availability statement

The datasets presented in this study can be found in online repositories. The names of the repository/repositories and accession number(s) can be found in the article/**Supplementary Material**.

## Ethics statement

The studies involving human participants were reviewed and approved by Department of Radiation Oncology, Shandong Cancer Hospital and Institute, Shandong First Medical University and Shandong Academy of Medical Sciences, Jinan, Shandong. The patients/participants provided their written informed consent to participate in this study.

## Author contributions

Equal contribution: CL and HS. First authorship: CL and HS. Equal contribution and senior authorship: WH, ZW, CF, DH, QZ, XW, and BL. All authors contributed to the article and approved the submitted version.

## Supplementary material

Supplementary Table 1 One-way ANOVA analysis.
