# Supplementary figures and images for: Sintilimab as maintenance treatment for local/regional recurrent esophageal squamous carcinoma after concurrent chemoradiotherapy: a single-arm Ib/II phase study
Source: Front Immunol. 2023 May 31;14:1193394. doi: 10.3389/fimmu.2023.1193394 (PMC10264589; doi:10.3389/fimmu.2023.1193394)

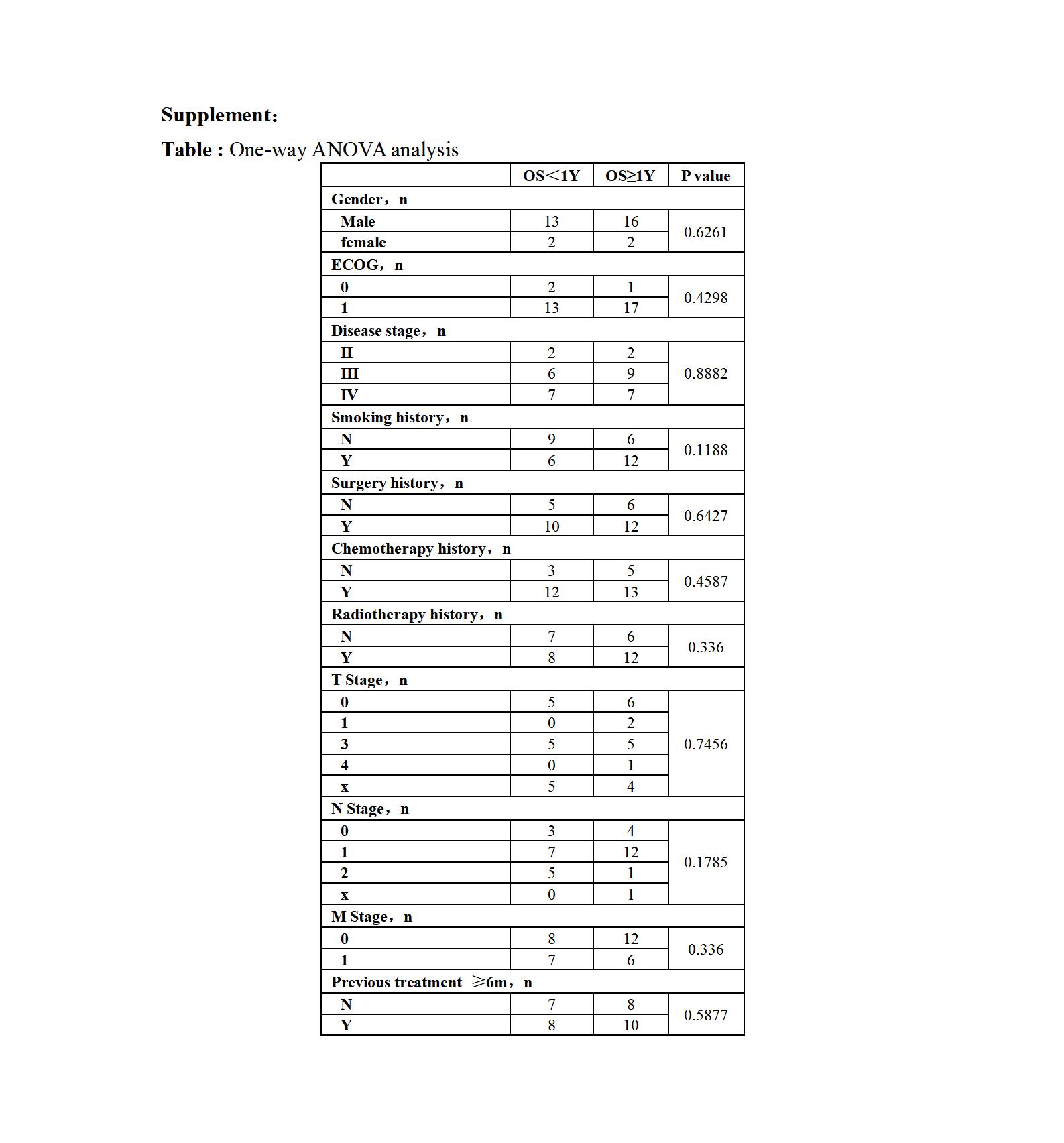

Supplement: Supplementary Table 1 — One-way ANOVA analysis. [file Image_1.jpeg]
